# Every OGT Is Illuminated … by Fluorescent and Synchrotron Lights

**DOI:** 10.3390/ijms18122613

**Published:** 2017-12-05

**Authors:** Riccardo Miggiano, Anna Valenti, Franca Rossi, Menico Rizzi, Giuseppe Perugino, Maria Ciaramella

**Affiliations:** 1 DSF-Dipartimento di Scienze del Farmaco, University of Piemonte Orientale, Via Bovio 6, 28100 Novara, Italy; riccardo.miggiano@uniupo.it (R.M.); franca.rossi@uniupo.it (F.R.); menico.rizzi@uniupo.it (M.R.); 2 Institute of Biosciences and BioResources, National Research Council of Italy, Via Pietro Castellino 111, 80131 Naples, Italy; anna.valenti@ibbr.cnr.it

**Keywords:** DNA repair, alkylation damage, conformational changes, protein structure, protein stability, protein-tag

## Abstract

*O*^6^-DNA-alkyl-guanine-DNA-alkyl-transferases (OGTs) are evolutionarily conserved, unique proteins that repair alkylation lesions in DNA in a single step reaction. Alkylating agents are environmental pollutants as well as by-products of cellular reactions, but are also very effective chemotherapeutic drugs. OGTs are major players in counteracting the effects of such agents, thus their action in turn affects genome integrity, survival of organisms under challenging conditions and response to chemotherapy. Numerous studies on OGTs from eukaryotes, bacteria and archaea have been reported, highlighting amazing features that make OGTs unique proteins in their reaction mechanism as well as post-reaction fate. This review reports recent functional and structural data on two prokaryotic OGTs, from the pathogenic bacterium *Mycobacterium tuberculosis* and the hyperthermophilic archaeon *Sulfolobus solfataricus*, respectively. These studies provided insight in the role of OGTs in the biology of these microorganisms, but also important hints useful to understand the general properties of this class of proteins.

## 1. Introduction

*O*^6^-DNA-alkyl-guanine-DNA-alkyl-transferases (EC: 2.1.1.63) are small (17–22 kDa) proteins that catalyze repair of DNA lesions induced by alkylating agents, a class of mutagenic and carcinogenic agents present in the environment. These proteins are evolutionarily conserved across the three domains of life, and are known by different acronyms (AGT, for alkyl-guanine-DNA-alkyl-transferase; OGT, for *O*^6^-alkyl-guanine-DNA-alkyl-transferase, or MGMT, for *O*^6^-methylguanine-DNA methyltransferase) in different organisms [1,2,3,4,5] and hereafter will be collectively indicated as OGTs. Alkylating agents can be divided into two subgroups, namely the SN_1_ and SN_2_ types respectively, which act with different mechanisms. Those belonging to the SN_1_ type (such as *N*-methyl-*N*-nitrosourea and *N*-methyl-*N*′-nitro-*N*-nitrosoguanidine) act by a monomolecular mechanism and can induce both *N*- and *O*-methylation, whereas the SN_2_ type drugs (such as methyl methanesulfonate (MMS) and methyl iodide) mainly induce *N*-methylation by bimolecular mechanisms [6]. Alkylation occurs preferentially at position *N*^7^ or *O*^6^ of guanine and *N*^3^ of adenine. Alkylation damage is particularly harmful for genomes because, if unrepaired, it can result in base mismatch, replication fork stalling, single or double strand breaks [6,7,8,9,10]. These lesions can be repaired by different enzymes. DNA glycosylases, such as AlkA and Tag remove *N*^3^-methyladenine (3meA) and create abasic sites in DNA, which are subsequently repaired by the base excision repair system (BER). Dioxygenases, such as AlkB, repair *N*^1^-methyladenine (1meA) and *N*^3^-methylcytosine (3meC) directly restoring the correct DNA sequence [6]. Finally, OGTs directly remove methyl groups from *O*^6^-methylguanine (*O*^6^meG) and *O*^4^-methylthymine (*O*^4^meT).

**Figure 1:**
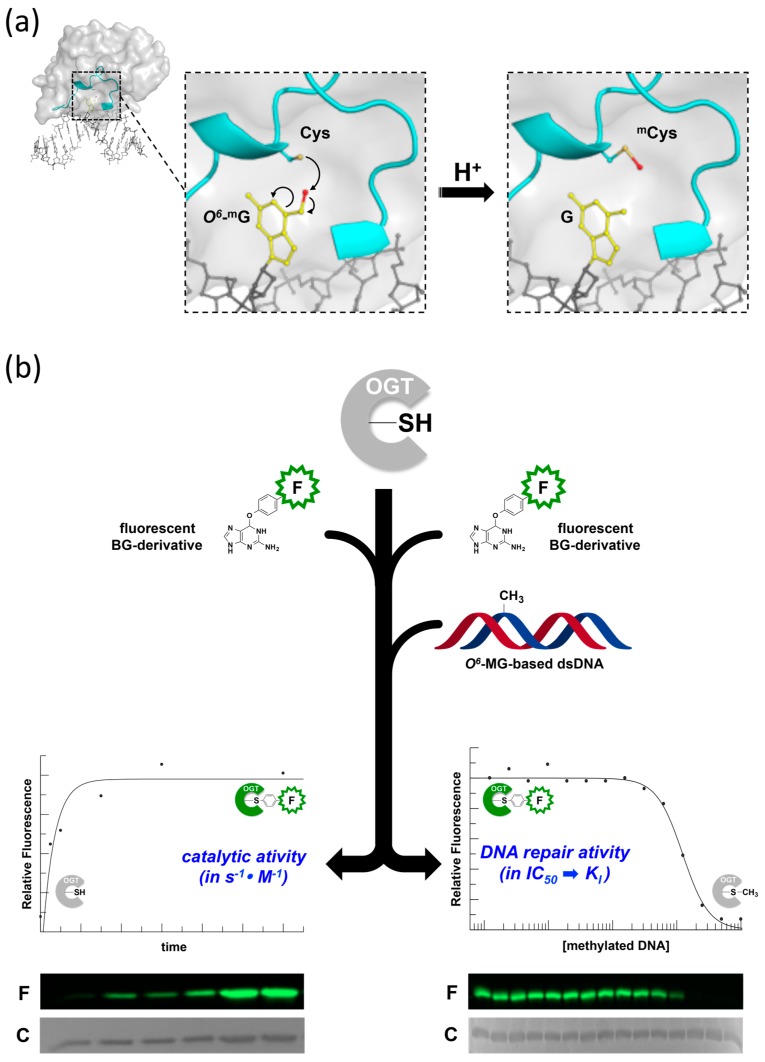
The DNA repair reaction by *O*^6^-DNA-alkyl-guanine-DNA-alkyl-transferases (OGTs). (**a**) Upon DNA binding, OGT flips-out the damaged guanine from the DNA backbone and irreversibly transfers the alkyl group to its own catalytic cysteine. (**b**) The covalent bond between the alkyl group and the cysteine allowed developing a new assay, by using *O*^6^-BG fluorescent derivatives alone (**left panel**), or in combination with natural non-fluorescent alkylated-DNA substrates (**right panel**).

OGTs are unique in their structure, mechanism and post-reaction outcome. They are able to perform whole repair reactions without the assistance of other factors or the need of energy source. OGTs are able to catalyze a trans-alkylation reaction, in which an alkyl group in DNA, mainly at position *O*^6^ of guanines or *O*^4^ of tymines, is transferred to a cysteine residue in the protein active site [3,4,11,12] (Figure 1a). This reaction is irreversible and determines the protein inactivation, thus each OGT molecule works only once [13]. The lesion recognition and repair on DNA occurs by a peculiar flipping-out mechanism, in which the protein extrudes the alkylated base form the double helix [4,12,14].

Historically, knowledge of OGTs was provided by classic studies on two proteins encoded by *Escherichia coli*, called Ada (adaptive response) and OGT [15,16,17]. In recent years, the human DNA-alkyl-transferase protein, called hAGT, raised a lot of interest because its overexpression in cancer cells has been associated with the onset of resistance to alkylating chemotherapy agents, making hAGT a potential therapeutic target [18,19,20]. In addition, hAGT has recently been the starting point for a new protein labelling strategy, called SNAP-tag^®^ technology [21,22,23].

In this review, we will focus on recent results reported on two prokaryotic OGTs, from the pathogenic bacterium *M. tuberculosis* (*Mt*OGT) and the hyperthermophilic archaeon *S. solfataricus* (*Ss*OGT), respectively. Among OGTs, these two proteins raised particular interest because of the potential involvement in virulence of the former and the peculiar thermal stability of the latter. In-deep biochemical characterization of both proteins has been performed, thanks to the development of novel assays and site-directed mutagenesis; moreover, a structural characterization of wild type and mutant versions of these two proteins allowed us to highlight important features of all OGTs. Finally, the peculiar thermostable nature of *Ss*OGT has allowed the development of a novel protein-tag for thermophilic organisms.

## 2. Development of Novel Alkyl-Transferase Assays

Although they are biocatalysts, OGTs are not conventional enzymes, as their reaction is irreversible and protein molecules are not recycled (Figure 1a). A number of assays have been used to measure OGTs activity; most of them rely on laborious and time-consuming procedures, implying the use of radiolabelled isotopes and/or chromatographic techniques, requiring high amount of purified proteins [24,25,26,27]. In order to overcome these limitations, novel DNA alkyl-transferase assays have been recently developed. These assays are based on the use of fluorescent derivatives of *O*^6^-benzyl-guanine (*O*^6^-BG), a competitive inhibitor of most OGTs. This molecule reacts with the catalytic cysteine of OGTs with the same mechanism as the natural substrate, resulting in covalent transfer of the benzyl group to the protein. If *O*^6^-BG is conjugated with a fluorogenic group, the latter is irreversibly transferred to the protein, with a one to one stoichiometry; hence the fluorescence intensity is a direct measure of the protein activity. The method can be applied to all OGTs sensitive to *O*^6^-BG inhibition, such as the human AGT and most bacterial and archaeal orthologs, and can be used under both native and denaturing conditions (Figure 1b, left side) [28]. The assay allows the determination of kinetics parameters for the trans-alkylation reaction [28,29]. In addition, if alkylated DNA is included in competition assays along with the fluorescent molecule, it is possible to measure the protein affinity for the natural substrate (Figure 1b, right side). The latter approach allows rapid determination of the half maximal inhibitory concentration (IC_50_), which can be converted to the dissociation constant for DNA (*K*_DNA_), giving an indirect measure of the efficiency of *O*^6^-MG repair by OGTs [28,29,30].

## 3. DNA Alkylation Damage and OGT-Mediated DNA Repair Response in *M. tuberculosis*

*M. tuberculosis* is an extremely well adapted human pathogen that spends the majority of its life inside the host in a non replicative state, confined to granulomas, where it inhabits the most inhospitable cells of the body: the infected macrophages. During its life cycle, *M. tuberculosis* is continuously exposed to DNA damaging stresses that could compromise the bacillary fitness during the different phases of the infection [31]. DNA damaging agents are mainly represented by potent DNA-alkylating chemical species, which originate by the action of reactive oxygen species (ROS), reactive nitrogen species (RNS) and other antimicrobial factors generated by the host immune system [32,33]. Similar to most bacteria, *M. tuberculosis* counteracts the deleterious effect of alkylating agents by mounting multi-enzymatic DNA repair response as well as by the expression of inducible genes of Ada response [34,35].

Although adaptive response is conserved among many bacterial species, the domains of Ada (namely AdaA and AdaB), AlkA and AlkB proteins exist in different combinations in different prokaryotes. In particular, *M. tuberculosis* shows a gene fusion of *adaA* with *alkA* (*adaA*-*alkA*), and an independent *adaB* gene (Tuberculist code: Rv1316c) also annotated as *ogt*, that encodes for the *Mt*OGT protein.

Gene silencing experiments demonstrated that *ogt* is not essential for infectivity and survival either in vitro or in the mouse model of *M. tuberculosis* infection [33,34,35,36]. However, several studies support the importance of OGT protein in protecting the mycobacterial GC-rich DNA from the promutagenic potential of *O*^6^-alkylguanine, occurring at different stage of the infection along *M. tuberculosis* bacilli exposure to different DNA-alkylating species. It was observed that the *ogt* gene expression undergoes a fine-tuning regulation during the infection and in response to alkylating agents [34,37,38]; moreover, the key role of *Mt*OGT protein in preserving mycobacterial genome from deleterious effects of alkylation damage is supported by trans-complementation experiment. Indeed, the heterologous expression of *Mt*OGT in the *E. coli* KT233 *ada*-*ogt*-defective strain suppresses its MNNG (*N*-methyl-*N*′-nitro-*N*-nitroso-guanidine) sensitivity [34] and rescues the hypermutator phenotype.

### 3.1. Biochemical and Structural Studies of M. tuberculosis OGT

*Mt*OGT, as its orthologs from other organisms, invariably performs the removal of alkyl adduct on modified guanines through a suicidal mechanism (Figure 1a), by performing the stoichiometric transfer of the *O*^6^-alkyl group to the strictly conserved cysteine residue in the protein active site, which is hosted in the C-terminal (C_ter_) domain along the -PCHR- signature protein sequence [29]. *Mt*OGT repairs methyl adducts on the *O*^6^-position of a guanine base in double-stranded DNA (dsDNA), as well as bulky adducts on isolated bases, as demonstrated by in vitro experiment using the fluorescent derivative of *O*^6^-BG (SNAP-Vista Green^®^) with a dissociation constant (*K*_VG_) in the low micromolar range [29]. On the contrary, the *E. coli* Ada and OGT are inactive on *O*^6^-BG adducts in dsDNA [39,40]. *Mt*OGT recognizes a double-stranded DNA fragment bearing an internal *O*^6^-methylated guanine with affinity ranking in the micromolar range; moreover, it binds dsDNA in a cooperative manner with a dissociation constant value (*K*_D_) of approximately 7.0 μM, a value comparable to that determined for hAGT by using the same method [41]. Recognition of a damaged site is the molecular determinant for the thermodynamic-driven alkyltransferase reaction with the protein that specifically recognizes damaged site upon a normal base that is transiently placed in the active site. Indeed, *Mt*OGT recognizes a modified dsDNA substrate with a 30-fold higher affinity with respect to unmodified DNA [29].

**Figure 2:**
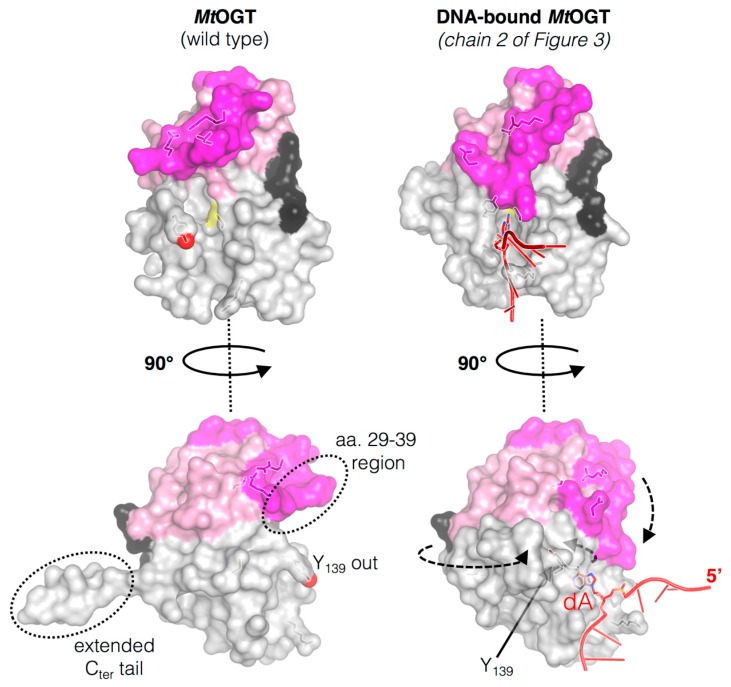
Structural rearrangements occurring on *M. tuberculosis* OGT (*Mt*OGT) in its ligand-free form (Protein Data Base, PDB ID: 4BHB) along DNA binding process (PDB ID: 4WX9); the regions affected by the conformational changes are indicated with dotted circle in ligand-free structure while the arrows indicate the direction of the movements of the N-terminal flap, the active site loop and the C-terminal tail of the protein as consequence of DNA binding (adapted from [45]).

Similar to structures of other prokaryotic OGTs, as well as of hAGT in different ligand-bound states [30,42,43,44], *Mt*OGT folds into a roughly globular molecular architecture built up by two domains connected by a long loop and ending in a 10-residue-long tail (Figure 2). The *Mt*OGT N-terminal domain (N_ter_) consists of an anti-parallel three-stranded β-sheet and connecting loops constrained between a mainly randomly coiled region, containing a single helical turn at its middle, on one side and a structurally conserved α-helix on the opposite one. The C_ter_ adopts the typical all-α-fold and houses the highly conserved elements that are functional to a proper reaction performing: (*i*) the helix-turn-helix (HTH) motif, which is involved in DNA binding at its minor groove and bears the arginine residue (R109) acting as a temporary steric substitute for the modified base upon its flipping-out from the regular base stacking; (*ii*) the “Asn hinge” contributing to the formation of the ligand binding pocket, that accepts the modified base; (*iii*) the catalytic cysteine (C126); (*iv*) the active-site loop that participates in the correct positioning of the alkylated base inside the ligand binding pocket; and (*v*) the structurally conserved H6 helix that, by building the ligand binding cavity on the opposite side of the Asn hinge, contributes essential residues for completing the modified-base bonding network [29,45].

### 3.2. The Active-Site Loop of MtOGT Is Intrinsically Flexible

The active site loop and the C_ter_ tail of *Mt*OGT adopt different conformations, depending on the association of the protein with the DNA substrate (Figure 2). The C-side region of the active site loop (residues 136–141) of the ligand-free structures of *Mt*OGT and of all the mutated variants characterized until now is invariably oriented towards the bulk solvent, in a so-called “unbound/out” conformation [29,45]. This unprecedented orientation of the active site loop is reminiscent of that observed for the same region in the *Methanococcus jannaschii* OGT structure in solution [43], but strongly differs from what was observed in other OGTs for which the crystal structure has been solved [14,30,42,46,47,48]. On the contrary, the C-side of the active site loop of each protein chain that builds up the *Mt*OGT::dsDNA complex structure is oriented inwards the active site pocket, where it participates in fitting the ligand-binding cavity to the flipped-out base [45]. These observations raise the possibility that the active site of *Mt*OGT could exist in two alternative conformations: “active site loop out” in the ligand free state or “active site loop in” in the DNA-bound form (Figure 2). These observations highlight the high degree of structural flexibility of this region in the mycobacterial protein with respect to the equivalent region of the well-characterized human ortholog.

Site-directed mutagenesis and structural studies demonstrated that the conserved Y139 residue of the active site loop of *Mt*OGT could play a role not only in properly fixing the base inside the protein active site [29], as proposed for hAGT Y158 [25,26,40,41,42], but also in making *Mt*OGT able to discriminate between intact and alkylated dsDNA molecules, albeit through a molecular mechanism that is still clear. In fact, the Y139F mutated variant appears less affected in its ability to bind unmodified dsDNA, while its affinity for the modified dsDNA is clearly negatively affected. In principle, the substitution of Y139 with a phenylalanine should have little effect on catalysis, since the capability of narrowing the ligand-binding pocket by providing an aromatic environment for the alkyl adduct remains unaltered [14,48].

**Figure 3:**
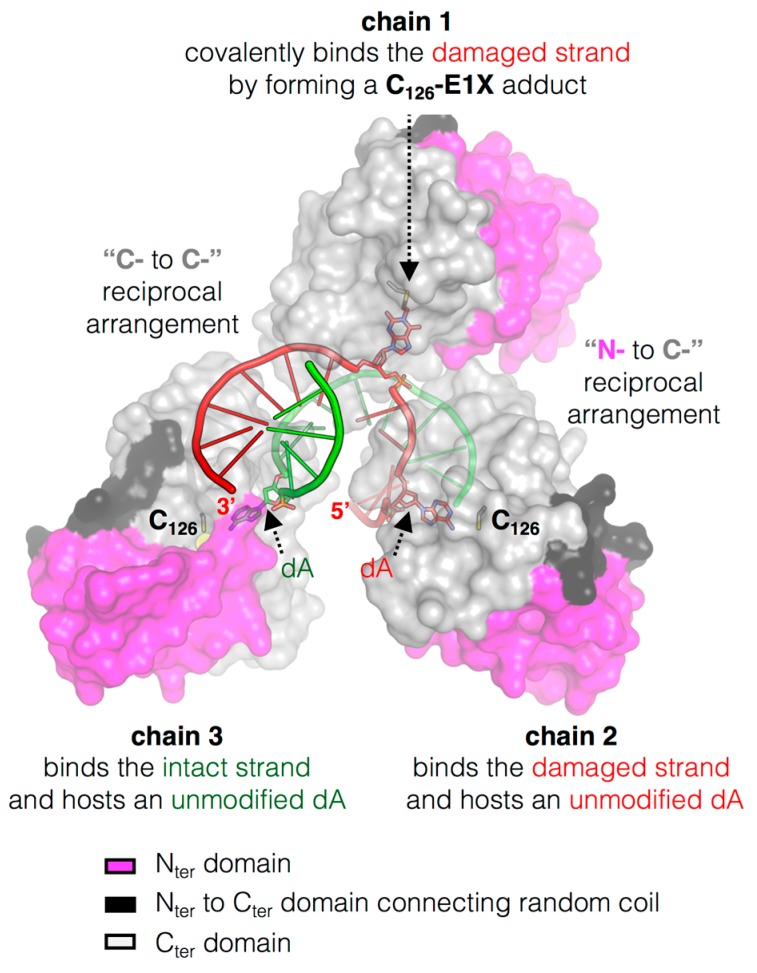
The peculiar cluster of *Mt*OGT. Surface representation of three *Mt*OGT units on an E1X-dsDNA which behaves as a mechanistic inhibitor of the protein (PDB ID: 4WX9); the two protein domains are depicted with different colors as indicated in the figure, the dsDNA molecule is rendered as cartoon and the extra-helical bases are rendered as sticks (adapted from [45]).

The high-resolution crystal structure of the recombinant *Mt*OGT R37L mutated variant, together with the biochemical analysis of highly homogeneous recombinant versions of *Mt*OGT T15S and *Mt*OGT R37L were reported [29]. *Mt*OGT R37L displayed a ten-fold reduced affinity towards methylated dsDNA, as compared with the wild-type protein, although the mutation did not affect the reaction rate. Moreover, the R37L aminoacidic substitution also affects the direct protein-DNA association, as it was observed in the electrophoretic mobility shift assay (EMSA)-based analysis using unmodified double-stranded oligonucleotides. The R37 residue is involved in the coordination of a peculiar network of bonds established between the core β-sheets and the facing random coil of the N_ter_ and its substitution could affect the conformational stability of such a region. Taking into account the defective DNA repair activity of the R37L mutated protein, it was suggested that the conformational stability of peculiar regions at the N_ter_ of the protein is of relevant importance for the catalysis taking place at the far C-terminal domain [29,45]. The analysis of the crystal structure of *Mt*OGT in complex with a modified dsDNA molecule, *N*^1^-*O*^6^-ethano-2′-deoxyxanthosine-containing dsDNA (*Mt*OGT::E1X-dsDNA, Figure 3; [45]) seems to exclude direct participation of R37 in DNA binding, because the protein residue and the sugar–phosphate backbone of the dsDNA substrate were at a distance of >16 Å. Instead, R37 could function as a hinge, limiting the conformational plasticity at the C-side of the flap of the N_ter_, by participating to keep it in contact with the bulky core of the N-terminal domain, upon the protein-DNA complex formation. In principle, the absence of such an anchoring site could affect the capability of the flap to undergo discrete movements and the resulting unconstrained flexibility of the N_ter_ random coil could hamper the correct assembly of *Mt*OGT cooperative clusters at the damaged DNA sites.

### 3.3. The N-Terminal Domain of MtOGT Attends DNA Cooperative Binding Process

A number of geographically distributed *M. tuberculosis* strains (like the W-Beijing strain and multi-drug resistant isolates) have been identified which carry non-synonymous SNPs in their ogt genes [49,50]. These point mutations result in aminoacid substitution at position 15 (*Mt*OGT T15S) or position 37 (*Mt*OGT R37L), mapping at the *Mt*OGT N-terminal domain. A limited number of studies put the focus on the OGTs N-terminal domain, which could play a role in the coordination of the catalytic cycle [11,46] and/or in mediating protein assembly at the site of damage upon DNA binding [11,14,51,52,53].

The high-resolution crystal structure of the recombinant *Mt*OGT R37L mutated variant, together with the biochemical analysis of highly homogeneous recombinant versions of *Mt*OGT T15S and *Mt*OGT R37L were reported [29]. *Mt*OGT R37L displayed a ten-fold reduced affinity towards methylated dsDNA, as compared with the wild-type protein, although the mutation did not affect the reaction rate. Moreover, the R37L substitution also affects the direct protein-DNA association, as it was observed in EMSA-based analysis using unmodified ds-oligonucleotides. The R37 residue is involved in the coordination of a peculiar network of bonds established between the core β-sheets and the facing random coil of the N-terminal domain; its substitution could affect the conformational stability of this region. Taking into account the defective DNA repair activity of the R37L mutated protein, it was suggested that the conformational stability of peculiar regions at the N-terminal domain of the protein is of relevant importance for the catalysis taking place at the far C-terminal domain [29,45]. The analysis of the crystal structure of *Mt*OGT in complex with a modified dsDNA molecule, *N*^1^-*O*^6^-ethano-2′-deoxyxanthosine-containing dsDNA (*Mt*OGT::dsDNA; [45]) (Figure 2) seems to exclude a direct participation of R37 in DNA binding, because the protein residue and the sugar–phosphate backbone of the dsDNA substrate were at a distance of >16 Å. Instead, R37 could function as a hinge, limiting the conformational plasticity at the C-side of the flap of the N-terminal domain, by participating to keep it in contact with the bulky core of the N-terminal domain, upon the protein-DNA complex formation. In principle, the absence of such an anchoring site could affect the capability of the flap to undergo discrete movements and the resulting unconstrained flexibility of the N-terminal domain random coil could hamper the correct assembly of *Mt*OGT cooperative clusters at the damaged DNA sites.

### 3.4. The Structure of MtOGT in Complex with Modified DNA Sheds Light on Cooperative Substrate Binding

The crystal structure of the *Mt*OGT::dsDNA complex revealed an unprecedented possible mode of assemblage of three adjacent protein chains onto the same damaged DNA duplex and could explain the cooperative DNA-binding mechanism of *Mt*OGT, which was demonstrated by EMSA-based analyses [29,45]. The association of *Mt*OGT with the dsDNA substrate induces the repositioning of three solvent-exposed protein regions (Figure 2): a random coiled segment (residues 29–39) of the N-terminal domain, part of the active site loop (residues 135–142) and the C-terminal tail (residues 156–165). Therefore, each protein monomer in the *Mt*OGT::dsDNA complex appears more compact than the ligand-free protein.

**Figure 4:**
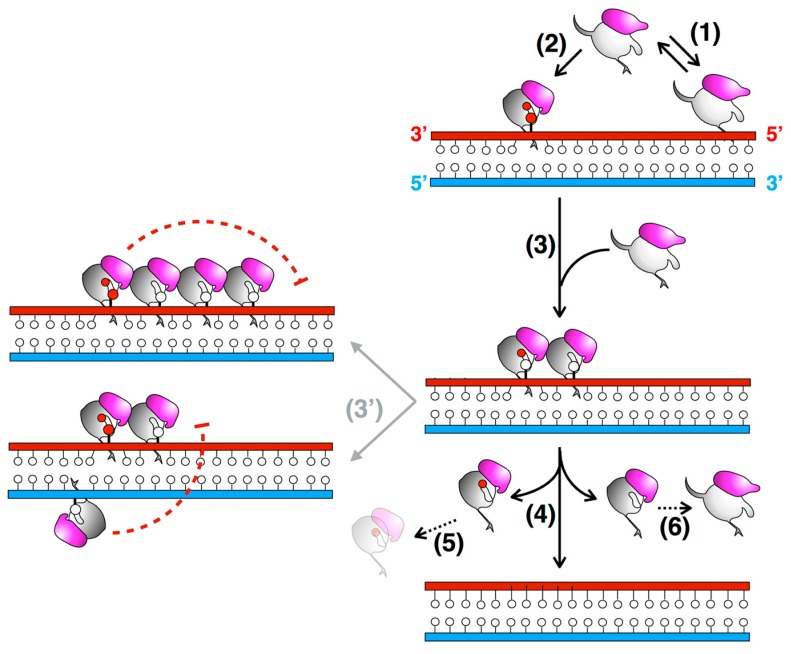
Proposed DNA repair mechanism involving *Mt*OGT. Sub-optimal OGT binding to undamaged dsDNA could result in protein-DNA complex dissociation (1); when a lesion is encountered, conformational modifications could take place (2); leading to the recruitment of additional OGT subunits tightly packed at a few bases apart, where they check the DNA for further modified bases (on both strands) (3); However, the formation of a continuous protein coat onto dsDNA could be hampered by long- and short-range steric hindrance phenomena (red dashed T-bars) (3′), until the protein-DNA complex disassembles upon repair (4); The final fate of alkylated MtOGT (5) and the capability of the released, un-reacted protein monomers to undergo further conformational changes (6) are still unknown (black dotted arrows) (adapted from [45]).

The architecture of *Mt*OGT as it was observed in the protein-DNA complex structure is a snapshot of a potential reaction step at which the modified base has been irreversibly bound by one protein unit, whereas two other subunits occlude available binding sites on both strands by flipping and housing in their active site the unmodified nucleobases (Figure 3). The *Mt*OGT protein cluster was more compact, if compared with the one proposed for hAGT [11,51,52,53,54], possibly due to the structural plasticity of *Mt*OGT, which could allow more crowded protein assembling onto DNA. Interestingly, the model built on further DNA-bound *Mt*OGT monomers towards the 5′-end of the modified strand, revealed that the association of a *Mt*OGT monomer with the region of the intact DNA strand facing the alkylated base hampers recruitment of additional protein subunits at the 5′-side of the damaged base. The analysis of a model built by omitting the protein chain bound to the undamaged strain revealed that the DNA binding-associated repositioning of the N_ter_ flap enables additional contacts between adjacent subunits. Finally, both short- and long-range steric hindrance phenomena could play a role in regulating *Mt*OGT-DNA association and dissociation, resulting in protein clusters that are capable of self-limiting their own size, similar to what has been experimentally determined by direct atomic force microscopy studies on human AGT (Figure 4) [55].

The cooperative assembly of protein-DNA complexes might contribute to the efficiency of lesion search and removal, due to the higher density of repair activity at the site of damage than that occurring in a non-cooperative DNA-binding mechanism [51]. Furthermore, the systematic scanning of DNA could be facilitated by the small and self-limiting *Mt*OGT protein clusters, allowing the alkylation damage search to be coupled with transcription and replication processes, as has been suggested for the human protein [55]. Moreover, an inherent capability of the protein to limit its own distribution on DNA could influence the rates of association to and dissociation from the target, and hence the kinetics of the lesion search; in fact, repositioning of a subunit placed in the middle of a single long protein cluster should probably be slower than repositioning of subunits mapping at the ends of many short clusters [51,55].

## 4. The *S. solfataricus* DNA Alkyl-Transferase, *Ss*OGT

Alkylating agents are widely present in the environment and are also produced by endogenous reactions, thus they pose a treat to the genome integrity of all cells. For thermophilic organisms, alkylation lesions are particularly harmful because high temperatures accelerate conversion of alkylated bases into DNA breaks, ultimately leading to DNA degradation [56]. Whereas OGTs are encoded by several thermophilic bacteria and archaea, limited information is available on these proteins [57,58,59]. Over the last few years, detailed biochemical, physiological and structural studies have been reported for the OGT protein from the hyperthermophilic archaeon *S. solfataricus* (called *Ss*OGT), a microorganism living in hot springs, at optimal temperature of 85 °C and pH 3.0. In order to maintain its genome stable under these highly challenging conditions, these organisms have evolved a number of very efficient repair and protection systems [28,30,60,61]. *Ss*OGT is a highly thermostable protein, which works under a variety of harsh conditions. Studies on this protein have been useful to understand its role in DNA damage response, elucidate structure-function relationships and describe conformational modifications occurring during the different steps of the reaction. The results have been useful to propose a general paradigm to correlate the structure and stability of OGTs with the active site status, which could be applied to many, if not all OGTs. Moreover, for its peculiar features, *Ss*OGT has been used to develop a useful protein-tag for protein imaging in thermophilic organisms [62,63].

### 4.1. Biochemical Characterization of SsOGT

Using a combination of the assays exploiting derivatives of *O*^6^-BG described in paragraph 2, the properties of *Ss*OGT heterologously expressed in *E. coli* were carefully characterized (Table 1). The protein showed activity over a wide range of conditions: in agreement with the thermophilic nature of *S. solfataricus*, *Ss*OGT optimal catalytic activity was at 80 °C, but it was also relatively active at temperatures as low as 25 °C; in addition, the protein showed significant activity in the pH 5.0–8.0 interval, was tolerant to a number of different reaction conditions, such as ionic strength, low concentrations of detergents, organic solvents and ethylenediaminetetraacetic acid (EDTA) [28], and was strikingly resistant to proteases [62].

*Ss*OGT binds DNA in a cooperative manner, and efficient binding depends on the arginine 102 residue and the HTH domain [28]. Indeed, mutation of the R102 residue reduces DNA binding efficiency, whereas mutation of up to five residues in the HTH motif completely abolishes the protein ability to form stable complexes with DNA, although the mutant is normally folded and able to perform the trans-alkylation reaction if *O*^6^-BG derivatives are used [28]. *Ss*OGT binds methylated oligonucleotides independently from the position of the lesion, but repair is position-dependent [29]: efficient repair requires the presence of at least 2 bases from the 5′ end and 4 bases from the 3′ end of the DNA molecule. This is likely due to the asymmetric interactions formed by the two protein sides with the double helix, as confirmed by structural models [30].

### 4.2. SsOGT and the S. solfataricus Response to Alkylating Agents

In line with the notion that combination of high temperature with alkylating agents exacerbates DNA damage, *S. solfataricus* is highly sensitive to drugs such as MMS [28,56,64]. Treatment of *S. solfataricus* cultures with relatively low concentrations of this agent induces an apoptotic-like phenomenon characterized by degradation of a number of proteins involved in DNA damage response, DNA fragmentation and cell death [28,56,64]. At lower MMS concentrations, cell growth is restored after a transient arrest. The single *ogt* gene of this species is dispensable for growth; indeed, a knock-out strain obtained by CRISPR (Clustered Regularly Interspaced Short Palindromic Repeats)-Cas9 (CRISPR associated protein 9) technology is viable and does not show significant growth impairment [63]. The *ogt* steady-state RNA level was increased after treatment with MMS, suggesting that the protein takes part in an inducible response to DNA damage [28]. Interestingly, despite the transcriptional induction, the *Ss*OGT protein is degraded in *S. solfataricus* cells in response to treatment with MMS, and degradation is triggered by alkylation of *Ss*OGT and some pathway activated in vivo in response to alkylation damage. Since a similar phenomenon occurs in human and yeast cells [13,65], these results suggest a striking evolutionary conservation, from archaea to higher eukaryotes, of an important repair system, as shown for all DNA metabolic processes [65]. In eukaryotes, OGT degradation is triggered by ubiquitination, which targets the alkylated protein to the proteasome [13,65]. Whereas ubiquitin is not present in archaea, ubiquitin-like small proteins have been found, along with a proteasome devoted to degradation of damaged proteins. It is thus possible that some post-translational modification might target the protein to degradation pathways, either directly or after interaction with other factors [66].

### 4.3. Crystal Structure of Free and DNA-Bound SsOGT

The crystal structure of *Ss*OGT, solved at 1.8 Å resolution showed a very well conserved folding with respect to the other proteins of the family, with the typical two domains joined by a connecting loop [30]. As for the latter protein, the N-terminal domain was less conserved, whereas higher conservation is found in the C-terminal domain, which contains all amino acid residues and structures important for both DNA binding and repair, including the active site loop with the catalytic C119 residue, the HTH motif for DNA binding and the so called “arginine finger” R102. An interesting feature, not present in other AGTs, is the C29-C31 S-S bridge of the N-terminal domain, which is an important structural element, contributing to the impressive thermal stability of *Ss*OGT (Table 2; [30]). Although disulfide bonds are rare in intracellular mesophilic proteins, recent genomic and biochemical data show that they are present in intracellular proteins of hyperthermophilic archaea, thus suggesting a role for disulfide bonding in stabilizing at least some thermostable proteins [67,68].

The structure of *Ss*OGT bound to methylated DNA was obtained thanks to the availability of an inactive C119A mutant, which is able to form a stable complex, but not to repair the alkylated base [30]. The crystal structure of the complex showed that each *Ss*OGT molecule occupies 4 base-pairs (bp) on dsDNA substrate. The protein forms several interactions at the 3′ side of the methylated base, which are important to stabilize the complex, confirming the results obtained in binding experiments, showing that at least 2 bases downstream to the *O*^6^-MG are needed to establish correct interactions, whereas at the 5′ end of the *O*^6^-MG two bases are sufficient for efficient repair.

**Figure 5:**
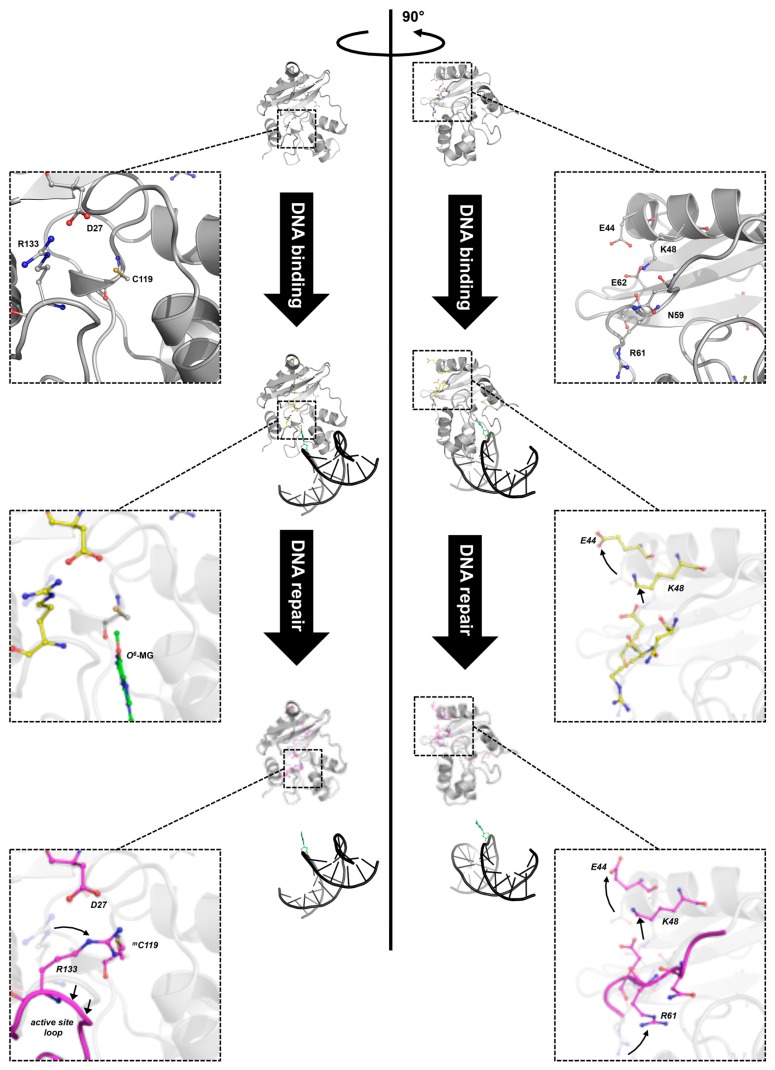
Different stages of DNA repair reaction of *Ss*OGT. The free form of *Ss*OGT (PDB ID: 4ZYE, in grey) recognizes and binds the alkylated DNA, leading to reversible conformational changes in its own structure (PDB ID: 4ZYD, in yellow), mainly involving the K48-network. Upon the alkyl transfer, the protein undergoes dramatic changes (PDB ID: 4ZYG, in magenta), which cause its inactivation and degradation. Insets: zoom-in of regions involved in the main conformational changes (adapted from [30,61]). Green, methylated guanine. Amino acid atoms are coloured according the CPK convention (carbon, in the corresponding colour of each 3D structure; oxygen, in red; nitrogen, in blue, sulphur, in yellow).

The crystal structure of *Ss*OGT::DNA complex showed that substrate binding does not affect significantly the protein overall structure [30]; however, structural changes in specific protein regions occur, in order to accommodate the double helix as well as the methylated base. Indeed, in silico mapping, performed by using difference distance matrix plots (DDMPs), of all interactions occurring in the molecule showed changes in the distance of a number of residues when the protein contacts DNA and the *O*^6^-MG is extruded into its active site [61]. These changes suggest that the molecule undergoes subtle movements, which might result in lost and gained interactions among residues forming intra-domain interactions. Moreover, important changes at the interface of the two domains are also observed upon DNA binding, involving a complex network of interacting aminoacid residues (K48, N59, R61 and E62 residues, called the K48-network). In the free *Ss*OGT, these residues are at a distance compatible with formation of an ionic/hydrogen bond network. Upon DNA binding, the E44 and K48 residues flip out, moving away from the protein core, leading to strong perturbation of the interaction network (Figure 5; [61]). As discussed in the next paragraph, these conformational changes are maintained in the post reaction form of the protein, when the active site is alkylated and DNA is released [61].

### 4.4. Alkylation of the Active Site Induces Dramatic Conformational Changes and Destabilization of SsOGT

Once the repair reaction has been completed, trans-alkylated OGTs undergo inactivation and dramatic destabilization. Thus, the efficiency of repair of alkylation lesions in vivo greatly depends on the OGT neosynthesis; this phenomenon has important consequences for the cell response to DNA damage as well as cancer treatments [3,20]. The structural mechanism of alkylation-induced destabilization is difficult to investigate exactly because once alkylated, OGTs unfold, aggregate and/or undergo degradation. Indeed, alkylated forms of the human protein could not be subjected to extensive biochemical analysis and were not stable enough to be crystallized. Structural information on alkylated hAGT could be obtained by incubating hAGT crystals in solutions containing alkylating agents; the presence of a methyl group could be accommodated in crystals, allowing resolution of the structure, which showed limited conformational rearrangements [14,46]. Bulkier alkyl adducts (such as a benzyl group) determined rapid crystal dissolution [46].

In contrast, although alkylation destabilized *Ss*OGT at its physiological temperature (>70 °C), at 25 °C the protein was enough stable to allow structural and biochemical analyses. In particular, resolution of the three-dimensional structure was obtained for the methylated form of *Ss*OGT, (*Ss*OGT^m^) obtained after incubation in solutions containing *O*^6^-MG, as well as of mutant proteins carrying substitution of the catalytic cysteine with either a leucine or a phenylalanine (C119L and C119F), which mimicked the presence of larger adducts in the active site (an isopropyl and a benzyl group, respectively). The structures of these three proteins showed extensive remodelling of interactions between aminoacid residues upon alkylation and large movements in the backbone structure [30,61]. These movements are determined by an active site expansion, as a consequence of binding of the alkyl group to the active site pocket, resulting in the increase of the distance between the active site loop and the recognition helix H4 of the HTH motif, which in turn affects the position of the adjacent structural elements (Figure 5).

In addition, structural and in silico analyses showed that significant changes in specific interdomain interactions occur at the interface between the N_ter_ and C_ter_ domains upon alkylation. One main change is observed at the level of D27 residue of the N_ter_ domain and R133 residue of the C_ter_ domain. Before reaction, these residues are at a distance compatible with ionic interaction; after alkylation, rotation of R133 could weaken or impair the interaction (Figure 5, left panel). Similarly, the H2 helix moves away from the protein core. Both modifications occur only upon alkylation, but not in the DNA-bound protein, thus suggesting that they are a direct consequence of the active site alkylation and not just substrate binding. In addition, the conformational changes observed in the protein-DNA complex at the interface between the N_ter_ domain and the connecting loop at the level of the K48-network are maintained after alkylation [61]. Thus, lesion recognition and alkylation trigger distinct modifications of intramolecular interactions in *Ss*OGT: whereas the changes at the level of the D27-R133 ion pair occur only upon irreversible trans-alkylation of the catalytic cysteine, those observed in the K48-network might be a consequence of lesion recognition/steric hindrance of the active site, since they are already found in protein in complex with the methylated DNA and are retained in the post-repair protein structure devoid of DNA (Figure 5, right panel) [61].

Biochemical and mutational analyses showed that alkylation of the active site leads to dramatic loss of stability of *Ss*OGT, (Table 2; [30,61]); whereas the Tm of wild type protein is 80 °C, a reduction of 17–35 °C is observed in variants were an adduct is present in the active site, and the extent of destabilization is linear with the hindrance of the adduct. Interestingly, a remarkable extent of destabilization is observed also in mutants where inter-domain interactions are perturbed, (Table 2) thus suggesting that the latter play important role in protein stability. On the basis of structure and biochemical data, it was suggested that the K48-network and D27-R133 ion pair are “locks”, which contribute to the correct folding of the protein in its free state. Active site/recognition helix conformational changes, which occur after binding to DNA and lesion recognition, determine opening of one “lock” (the K48-network). However, this modification is reversible until the C119 trans-alkylation occurs; once this reaction is completed, a second set of conformational changes determines the opening of the second “lock”, thus resulting in irreversible protein destabilization (Figure 5; [61]).

The specific inter-domain interactions which affect *Ss*OGT stability are not conserved in the structures of other OGTs. However, it is likely that the connection between the two domains play an important role in all OGTs. In the case of the protein from *Thermococcus kodakaraensis*, an inter domain ion pair network is important for the protein stability [42,69]. In hAGT, perturbation of interactions at the interface between the two protein domains is observed after alkylation, which resulted in dramatic hAGT destabilization [70]. These observations suggested a possible general model for OGTs, in which alkylation of the active site determines conformational changes at the level of the active site as well as of the interactions between the two domains, which trigger protein destabilization [30,61].

## 5. Development of OGT-Based Novel *Protein-Tags*

In recent years several *protein-tags*, which can be fused to proteins of interest to allow their detection and analysis, have been used in a number of organisms, enabling a wide variety of biological studies. These include the popular Green Fluorescent Protein (GFP) and its derivatives, which are intrinsically fluorescent [71,72], as well as proteins that can be labelled by an external substrate. In this context, a modified version of the hAGT, called SNAP-tag^®^, which is impaired in DNA binding and of higher operational stability, has been developed [21,22,23]. The advantage of the system consists in the possibility of labelling the chimeric protein with virtually any chemical group (such as a fluorophore, biotin, and so on), provided the latter is linked to the *O*^6^-BG molecule. This approach proved to be a highly specific and versatile tool for in vivo and in vitro specific labelling of proteins [21,22,23].

Thanks to its thermostable nature, *Ss*OGT was used to develop a version of the SNAP-tag^®^ protein suitable for thermophilic microorganisms. To this aim, a modified version of *Ss*OGT was used, in which DNA binding was abolished by the introduction of five mutations in the HTH domain [28]. This protein, called H^5^, was successfully used as a protein-tag in both mesophilic (*E. coli*) and thermophilic microorganisms (the bacterium *Thermus thermophilus* and the archaeon *Sulfolobus islandicus*). Plasmids expressing fusions of the H^5^-tag with two thermostable proteins were constructed, namely the *S. solfataricus* β-glycosidase and the thermophile-specific DNA topoisomerase reverse gyrase [62,73,74,75,76]. Both proteins were correctly expressed, folded, functional and stable, when the expression plasmids were introduced by transformation in *T. thermophilus* and *S. islandicus*, respectively. The presence and the activity of the H^5^-tag could be imaged in living cells as well as in cell-free protein extracts [62,63]. In addition, the H^5^-tag did not interfere with the enzyme activity of target proteins and could be fused to thermostable proteins overexpressed in *E. coli*, allowing to perform purification protocols including thermal treatment to precipitate aggregated host proteins [62].

The H^5^-tag might be used as a thermostable version of the SNAP-tag^®^ protein in other (hyper)thermophilic archaea and bacteria allowing detection and sub-cellular localization of proteins and protein interactions. In addition, the use of different fluorescent ligands could be used to label different proteins, including in pulse-chase analysis, and follow their movements and fate in the cell in real time. Both experiments were performed in the absence of endogenous OGT activity; indeed, *T. thermophilus* is a natural *ogt*^(−)^ species [62,77], whereas a *S. islandicus* mutant strain deleted for the *ogt* gene obtained by a CRISPR-based genome-editing method was used as a host [63]. Thus, it remains to be determined whether possible alkyl-transferase background activity might interfere with the efficacy of the system.

## 6. Conclusions and Perspective

Despite their completely different lifestyle, both *M. tuberculosis* and *S. solfataricus* face challenging external conditions, where alkylation damage is a serious treat for their genome integrity. Thus, studies on *Mt*OGT and *Ss*OGT help in understanding the function of these proteins in the biology of both microorganisms and their protection mechanisms. In addition, these proteins are useful models to elucidate the role of each structural element in the different OGTs activities, the details of DNA binding and lesion recognition, as well as the conformational changes associated with each step of the reaction. These results could have a wide impact, providing a general picture of how OGTs work, which is the foundation for structure-based design of novel OGTs inhibitors to be used in cancer and possibly other pathological conditions.

## Figures and Tables

**Table 1:** Biochemical properties of *S. solfataricus* OGT (*Ss*OGT), using SNAP-Vista Green^®^ as substrate (data are from [28,30]).

T_opt_		80 °C
Activity	25 °C	25%
	37 °C	45%
	80 °C	100%
pH_opt_		6.0
catalytic activity (M^−1^ s^−1^)	25 °C	0.28 ± 0.03 × 10^4^
	70 °C	5.33 ± 1.49 × 10^4^
thermal stability (t_½_, min)	60 °C	257.2 ± 10.3
	70 °C	165.1 ± 16.5
	80 °C	18.7 ± 2.0
NaCl > 1.0 M		100% activity
EDTA > 10.0 mM		100% activity
Dithiotreithol (DTT)		not required
*O*^6^-MG-DNA (*K_i_*, µM)	close to 5′ end	4.29 ± 0.39
	central	0.83 ± 0.02
	close to 3′ end	56.6 ± 23.8

**Table 2:** Analysis of the thermal stability of *Ss*OGT and related mutants, by using Differential Scanning Fluorimetry. *Ss*OGT C119^m^ is the *Ss*OGT protein methylated by incubation in the presence of *O*^6^-methyl-guanine; *Ss*OGT H^5^ is a mutant carrying five mutations in the HTH domain (data from [30,61]).

Type	Mutation	∆T_m_ (°C)
-	*Ss*OGT	^a^
disulphide bond	*Ss*OGT C29A	−17.1
catalytic cysteine	*Ss*OGT C119^m^	−17.3
	*Ss*OGT C119L	−35.4
	*Ss*OGT C119F	−35.1
N_ter_-C_ter_ ion pair	*Ss*OGT D27A	−8.0
	*Ss*OGT D27K	−35.3
N_ter_-loop network	*Ss*OGT E44L	+0.7
	*Ss*OGT K48A	−8.0
	*Ss*OGT K48L	−9.6
DNA binding	*Ss*OGT H^5^	−5.0

^a^ Wild type *Ss*OGT exhibited a T_m_ of 80.0 °C.
